# Crosstalk Between the NRF2 Signaling Pathway and Heat Shock Proteins: Implications for Cellular Stress Response and Disease Therapy

**DOI:** 10.1155/omcl/8809922

**Published:** 2026-10-12

**Authors:** Milad Faraji, Saeed Nazifi

**Affiliations:** ^1^ Department of Basic Sciences School of Veterinary Medicine Shiraz University Shiraz Iran shirazu.ac.ir; ^2^ Department of Clinical Sciences School of Veterinary Medicine Shiraz University Shiraz Iran shirazu.ac.ir

**Keywords:** cellular stress response, heat shock proteins, HSF1, NRF2, proteostasis, redox homeostasis, tumor immunity

## Abstract

Cells are continuously exposed to oxidative and proteotoxic stress that disrupt redox balance and protein homeostasis. Two major cytoprotective systems—the nuclear factor erythroid 2–related factor 2 (NRF2) pathway and the heat shock response (HSR) mediated by heat shock factor 1 (HSF1) and heat shock proteins (HSPs)—form an interconnected stress‐adaptation network that coordinates redox defense, proteostasis, and cellular survival. Although historically viewed as largely independent pathways, accumulating evidence demonstrates extensive bidirectional and context‐dependent crosstalk through transcriptional coregulation, protein–protein interactions, posttranslational mechanisms, and shared molecular hubs, including p62/SQSTM1 and the ubiquitin–proteasome system. This network integrates antioxidant defense with protein quality control, mitochondrial homeostasis, and inflammatory and metabolic signaling; however, NRF2 and HSR responses can also become functionally uncoupled under specific biochemical and metabolic conditions. This review critically examines the molecular mechanisms underlying NRF2–HSP crosstalk and its functional consequences in neurodegeneration, cancer, ischemia–reperfusion (I/R) injury, aging, and tumor immunity. Particular attention is given to the dual role of this axis: transient activation promotes cytoprotection, proteostasis, and tissue recovery, whereas chronic or constitutive activation can support tumor metabolism, immune evasion, and therapeutic resistance. In I/R injury, endoplasmic reticulum (ER) stress (ERS)‐induced XBP1–HRD1 signaling may impose an additional constraint on NRF2 activity, highlighting an important limitation of endogenous cytoprotection. Finally, we discuss therapeutic strategies targeting NRF2, HSF1, and HSPs, emphasizing the challenges of systemic pathway modulation, the importance of pharmacodynamic biomarkers and molecular stratification, and the potential of rational combination approaches. A context‐, tissue‐, and cell‐specific understanding of NRF2–HSP signaling will be essential for translating this complex stress‐response network into precision therapeutic strategies.

## 1. Introduction

Cells are continuously exposed to a broad spectrum of endogenous and exogenous stressors, including reactive oxygen species (ROS), electrophilic compounds, metabolic disturbances, and proteotoxic insults, all of which threaten the maintenance of cellular redox balance and protein homeostasis (proteostasis) [1, 2]. To preserve cellular integrity, organisms have evolved highly conserved adaptive defense systems that detect stress signals, activate protective transcriptional programs, and restore damaged molecular networks [3]. Among these mechanisms, the nuclear factor erythroid 2–related factor 2 (NRF2) pathway and the heat shock response (HSR) represent two major and evolutionarily conserved cytoprotective networks responsible for coordinating antioxidant defense [4] and proteostasis maintenance [5], respectively.

The molecular foundations of NRF2 signaling were established through seminal studies by Yamamoto et al., which established NRF2 as a member of the cap’n’collar (CNC) basic leucine zipper (CNC‐bZIP) family of transcription factors and demonstrated its role in regulating antioxidant and cytoprotective gene expression [6, 7]. Subsequent investigations by Kensler [8] and collaborators highlighted the physiological and toxicological importance of the Kelch‐like ECH‐associated protein 1 (KEAP1)–NRF2–antioxidant response element (ARE) pathway in cellular protection against environmental electrophiles and carcinogens. The identification of KEAP1 as a cysteine‐rich substrate adaptor of the Cullin 3 (CUL3)‐RBX1 E3 ubiquitin ligase complex revealed the central mechanism controlling NRF2 stability under basal conditions [9]. Extensive biochemical studies by Dinkova‐Kostova et al. further demonstrated that electrophilic NRF2 activators modify specific reactive cysteine residues within KEAP1, including Cys151, Cys273, and Cys288, thereby altering KEAP1 conformation and impairing NRF2 ubiquitination and proteasomal degradation [10, 11]. Stabilized NRF2 subsequently accumulates in the nucleus, where it binds AREs and activates a broad cytoprotective gene battery involved in antioxidant defense, glutathione (GSH) metabolism, xenobiotic detoxification, mitochondrial protection, and cellular repair [12].

In parallel, the HSR represents a highly conserved proteostasis network that protects cells against protein misfolding, aggregation, and proteotoxic damage caused by thermal, oxidative, metabolic, and inflammatory challenges [13]. Seminal studies by Morimoto et al. established heat shock factor 1 (HSF1) as the master transcriptional regulator of the mammalian HSR, controlling the expression of molecular chaperones, including HSP70, HSP90, and small heat shock proteins (sHSPs) [14, 15]. Under physiological conditions, HSF1 remains predominantly inactive through association with chaperone complexes; however, upon proteotoxic stress, HSF1 undergoes activation, nuclear translocation, DNA binding, and transcriptional induction of HSP genes, thereby promoting protein folding, refolding, and degradation of damaged proteins [13].

Historically, NRF2‐mediated antioxidant signaling and the HSR were considered largely independent stress‐adaptation pathways, with NRF2 primarily governing redox regulation and the HSR controlling protein quality control. However, accumulating evidence now indicates that these systems are functionally interconnected and form an integrated stress‐response network. NRF2 activation has been reported to influence selected components of the HSR, including HSF1‐associated stress programs and specific HSP genes, although the magnitude and direction of this regulation appear to be highly dependent on the cellular context, tissue type, and the nature of the stress stimulus [16, 17]. Conversely, molecular chaperones such as HSP90 [18] and HSP70 [19] have been implicated in the regulation of NRF2 stability, localization, and transcriptional activity through mechanisms involving modulation of NRF2 turnover, KEAP1‐associated regulation, and the proteostasis machinery [20]. The functional integration of NRF2 and HSR pathways provides cells with a coordinated defense system capable of simultaneously restoring the redox equilibrium and maintaining protein homeostasis. This interaction is particularly relevant under complex pathological conditions in which oxidative stress and proteotoxic stress occur concurrently, including neurodegenerative disorders, cancer, ischemia–reperfusion (I/R) injury, chronic inflammation, and aging.

In this review, we systematically examine the biochemical and molecular mechanisms underlying NRF2–HSP crosstalk, including transcriptional regulation, protein–protein interactions, shared proteostasis regulators, and functional integration in cellular physiology. We further critically evaluate the contribution of this network to neurodegeneration, cancer, I/R injury, and aging, with particular emphasis on disease‐specific mechanisms, immunometabolic regulation, and tumor immune escape. Finally, we discuss translational approaches targeting the NRF2–HSP axis, including pathway activation strategies for cytoprotection, HSF1/HSP inhibition for cancer therapy, biomarker‐guided patient selection, and emerging opportunities for precision medicine.

## 2. The NRF2 Signaling Pathway: Master Regulator of Redox and Cellular Homeostasis

The NRF2, which is encoded by the NFE2L2 gene, is a transcription factor belonging to the CNC‐bZIP family [21]. It is broadly recognized as the primary orchestrator of the cellular antioxidant and cytoprotective response to a variety of endogenous and exogenous stressors [22]. The NRF2 protein governs the transcriptional induction of a large battery of cytoprotective genes, estimated to include over 250 different targets [23]. This regulation is achieved through its binding to a specific cis‐acting enhancer sequence known as the ARE, which has a core consensus sequence of 5^′^‐TGACnnnGC‐3^′^ [24]. The collection of genes regulated by this interaction encodes proteins involved in a wide array of defense mechanisms, including direct antioxidant enzymes, proteins for GSH synthesis and regeneration, and enzymes for detoxification [25]. Due to this extensive regulatory network, the NRF2 pathway is now understood to be a master regulator of cellular homeostasis, extending its influence beyond redox balance to modulate inflammation, metabolism, and proteostasis.

### 2.1. Canonical Regulation of NRF2 by the Keap1–Cul3–Rbx1 E3 Ligase Complex

Under physiological, unstressed conditions, the intracellular activity of NRF2 is maintained at a very low level through a continuous cycle of synthesis and degradation [26, 27]. The principal regulator of NRF2 stability is the KEAP1, a dimeric protein that functions as a substrate adaptor for a CUL3‐based E3 ubiquitin ligase complex [4]. Keap1 functions both as the substrate adaptor of the CUL3‐RBX1 E3 ubiquitin ligase complex and as a redox/electrophile sensor through its reactive cysteine residues [28]. The physical interaction between the NRF2 and Keap1 proteins is governed by a well‐defined “hinge‐and‐latch” mechanism. This model involves a high–affinity interaction where the ETGE motif of NRF2 binds to the Kelch domain of one Keap1 monomer (the “hinge"), followed by a lower‐affinity binding of the DLG motif to the second Keap1 monomer (the “latch") [29]. This specific conformational lock correctly positions NRF2’s Neh2 domain for polyubiquitination by the CUL3‐RBX1 machinery [30]. Subsequently, the polyubiquitinated NRF2 is targeted for rapid degradation by the 26S proteasome [31], resulting in a protein half‐life of ~20 min in unstressed cells [32, 33]. This elegant system ensures that the powerful NRF2‐driven transcriptional program remains tightly suppressed until a stress signal is detected.

### 2.2. Activation of NRF2: The Cysteine Thiol Code

The canonical activation of NRF2 is triggered by the disruption of its interaction with Keap1, which effectively halts its proteasomal degradation [34]. This disruption is primarily mediated by the modification of Keap1’s highly reactive cysteine thiols by various electrophiles and ROS [35]. These modifications induce a conformational change in the Keap1 protein, which compromises the low‐affinity DLG “latch” interaction, thereby preventing the efficient ubiquitination of NRF2 [29]. Specific cysteine residues within Keap1, often referred to as a “cysteine code,” exhibit differential reactivity to various stressors, allowing the cell to mount a tailored response [36]. For instance, Cys151 is a key target for many small‐molecule electrophiles, while Cys273 and Cys288 are critical for sensing peroxide‐induced stress [37]. Once stabilized, NRF2 accumulates in the cytoplasm and translocates to the nucleus. Inside the nucleus, NRF2 forms a functional heterodimer with small Maf (sMaf) proteins, the complex required to bind ARE sequences in target gene promoters and initiate transcription [38].

### 2.3. Noncanonical, Keap1‐Independent NRF2 Regulation

While the Keap1‐centric model is dominant, it is now clear that NRF2 activity is modulated by a more complex network of Keap1‐independent mechanisms, adding further layers of regulatory control. Among the most significant of these is the regulation by the multifunctional autophagy adaptor protein, p62/SQSTM1. The p62 protein contains a Keap1‐interacting region (KIR) with a STGE motif, which mimics the ETGE motif of NRF2 and allows it to competitively bind to the same site on Keap1 [39]. Under conditions of impaired autophagy, p62 accumulates, leading to the sequestration of Keap1 and resulting in sustained, noncanonical NRF2 activation [40]. This interaction establishes a critical positive feedback loop, as the gene for p62 (SQSTM1) is itself a transcriptional target of NRF2, thereby directly linking the cell’s proteostatic status to its antioxidant capacity [41, 42]. Beyond p62, other proteins can also directly influence NRF2 stability; for instance, the tumor suppressor p21 has been shown to compete with Keap1 for NRF2 binding [43]. Furthermore, posttranslational modifications (PTMs) directly on the NRF2 protein, such as phosphorylation by glycogen synthase kinase 3β (GSK‐3β), can promote its recognition by the β‐TrCP E3 ligase, leading to its degradation in a Keap1‐independent manner [44]. Importantly, these Keap1‐independent mechanisms highlight that NRF2 activity is closely coupled to cellular proteostasis, providing a mechanistic basis for its functional intersection with the HSR and molecular chaperone networks, as discussed below.

### 2.4. The NRF2‐ARE Gene Battery: A Multifunctional Defense System

The downstream transcriptional program activated by NRF2 is remarkably diverse, orchestrating a multifaceted cytoprotective response [45]. This “NRF2‐ARE gene battery” can be broadly categorized into several functional classes. A primary function is the maintenance of GSH homeostasis, achieved by upregulating genes for both GSH synthesis (e.g., GCLC and GCLM) and regeneration (e.g., GSR) [46]. NRF2 also fortifies the thioredoxin antioxidant system by inducing the expression of thioredoxin (TXN/TRX1) and thioredoxin reductase 1 (TXNRD1/TRXR1) [47]. In parallel, it drives the expression of canonical Phase II detoxification enzymes and direct antioxidant proteins, including NAD(P)H:quinone oxidoreductase 1 (NQO1) and heme oxygenase‐1 (HMOX1) [48]. Critically, NRF2 directly links redox balance to proteostasis by controlling the expression of genes for proteasome subunits (e.g., PSMB5) and the autophagy receptor p62/SQSTM1 [1]. Finally, NRF2 orchestrates metabolic reprograming to support its antioxidant functions, most notably by shunting glucose into the pentose phosphate pathway (PPP) via upregulation of enzymes like glucose‐6‐phosphate dehydrogenase (G6PD) to generate essential NADPH [49]. Collectively, the NRF2–ARE transcriptional program therefore extends beyond conventional antioxidant defense to encompass GSH metabolism, NADPH production, detoxification, autophagy, and proteasomal proteostasis. This broader functional scope provides an important mechanistic framework for understanding how NRF2 intersects with the HSF1–HSP network to coordinate redox control with protein quality maintenance.

## 3. The HSR: Guardians of Proteostasis

The maintenance of a functional proteome, a state known as protein homeostasis or proteostasis, is a fundamental prerequisite for cellular health and survival [50]. Cells are perpetually exposed to a variety of intrinsic and extrinsic stressors that can compromise protein structure, leading to misfolding and the formation of potentially cytotoxic protein aggregates [51]. To combat these threats, eukaryotic cells have evolved a highly conserved transcriptional program known as the [52]. The HSR functions as the primary defense mechanism against proteotoxic stress by inducing the rapid expression of a suite of cytoprotective genes, most notably those encoding HSPs [53]. These HSPs act as molecular chaperones, forming a sophisticated network that actively manages the proteome to ensure its integrity [54–56].

### 3.1. HSF1: The Master Transcriptional Regulator

In eukaryotic cells, the central orchestrator of the HSR is the transcription factor HSF1 [57]. Under physiological conditions, HSF1 is maintained predominantly in an inactive state through dynamic interactions with molecular chaperones and regulatory proteins, with continuous nucleocytoplasmic shuttling [58]. Its activity is actively repressed through a dynamic, multichaperone complex that primarily includes HSP90, HSP70, and the cochaperone p23 [59]. This inhibitory interaction is critical for preventing spurious HSR activation. HSP90, together with HSP70 and associated cochaperones, contributes to maintaining HSF1 in a repressed state, whereas HSP70 has a particularly important role in feedback‐mediated attenuation of HSF1 activity during stress recovery [58]. The activation of HSF1 is triggered by the accumulation of non‐native proteins, which act as a direct signal of proteotoxic distress [60]. These aberrant substrates sequester HSP70 from the inhibitory HSF1–chaperone complex, thereby releasing HSF1 from latency control and enabling its trimerization and transcriptional activation [61].

### 3.2. HSF1 Activation Cascade and HSP Effector Functions

Once released from its inhibitory complex, HSF1 undergoes a rapid, multistep activation process [58]. This cascade begins with its assembly into a DNA‐binding‐competent homotrimer, a process mediated by its N‐terminal leucine zipper domains [62]. The trimerization is followed by a series of extensive PTMs, of which stress‐induced hyperphosphorylation is considered critical for acquiring full transcriptional competence [63]. This fully activated HSF1 trimer then translocates into the nucleus and binds with high affinity to conserved DNA sequences known as heat shock elements (HSEs), located in the promoter regions of its target genes [64]. The canonical HSE architecture consists of at least three inverted repeats of the 5^′^‐nGAAn‐3^′^ motif, which serves as the docking site for the HSF1 trimer to recruit the transcriptional machinery [65].

The primary effectors of this response are the HSPs, a diverse group of chaperones that manage the proteome. The ATP‐dependent HSP70 family (e.g., HSPA1A) is a highly versatile group of “foldases” that recognize exposed hydrophobic regions on nascent or misfolded polypeptides, preventing their aggregation and facilitating their refolding with the assistance of HSP40 (DnaJ) cochaperones [66]. Critically, HSP70 also functions as a triage hub; when a protein is terminally damaged, HSP70 collaborates with the E3 ubiquitin ligase CHIP (C‐terminus of HSC70‐interacting protein) to tag the client for degradation by the proteasome [67]. The HSP90 family comprises ATP‐dependent molecular chaperones specialized in the conformational maturation and stabilization of a wide range of client proteins, including essential signaling kinases and transcription factors [68]. In contrast, small HSPs, such as HSP27 (HSPB1), are ATP‐independent “holdases” that form large oligomers to sequester unfolding proteins, thereby preventing their irreversible aggregation until they can be addressed by the ATP‐dependent refolding machinery [69] (Table 1).

**Table 1:** Major heat shock protein families, their proteostatic functions, and their relevance to NRF2–HSR crosstalk.

HSP family	Representative proteins	ATP dependence	Primary function	Key relevance to NRF2	Cellular localization
HSP70	HSPA1A, HSPA8	+	Folding/refolding; triage; degradation	NRF2 stability; CHIP‐associated proteostasis; HSF1 regulation	Cytoplasm, nucleus
HSP90	HSP90AA1, HSP90AB1	+	Client maturation and stabilization	NRF2 stability; indirect regulation via GSK‐3β	Cytoplasm, nucleus
Small HSPs	HSPB1/HSP27, CRYAB	−	Aggregation prevention/holdase activity	NRF2 stabilization and potential KEAP1‐independent regulation	Cytoplasm, nucleus
Mitochondrial HSPs	HSPA9/mtHSP70, HSPD1/HSP60	+	Protein import, folding and mitochondrial proteostasis	Indirect coordination with NRF2‐mediated redox and mitochondrial quality control	Mitochondria

*Note*: ATP dependence refers to the requirement for ATP hydrolysis during the chaperone cycle; small HSPs primarily function as ATP, independent holdases. The NRF2 relevance column summarizes established or context‐dependent interactions described in the literature and does not imply equivalent mechanistic strength across HSP families. HSPA1A, heat shock protein family A member 1A; HSPA8, HSP70 family member 8; HSP90AA1, heat shock protein HSPA1A, heat shock protein family A member 1A; HSPA8, heat shock cognate 71 kDa protein; HSP90AA1, heat shock protein 90α family class A member 1; HSP90AB1, heat shock protein 90 beta family member 1; HSPB1, heat shock protein family B member 1 (HSP27); CRYAB, αB, crystallin; HSPD1, heat shock protein family D member 1 (HSP60); HSPA9, heat shock protein family A member 9 (mitochondrial HSP70); NRF2, nuclear factor erythroid 2–related factor 2; HSR, heat shock response; KEAP1, Kelch‐like ECH, associated protein 1; CHIP, C, terminus of HSC70‐interacting protein.

### 3.3. Attenuation of the HSR

The HSR is a potent but transient program that must be precisely terminated to restore cellular homeostasis. The attenuation of the response is governed by a classic negative feedback loop that is integral to the system’s design [70]. As the newly synthesized HSPs, particularly HSP70 and HSP90, accumulate to high levels, they first work to resolve the proteotoxic stress by refolding or degrading the backlog of damaged proteins [71]. Once the concentration of misfolded substrates decreases, the chaperones are free to reassociate with the activated HSF1 trimer [72]. This binding promotes the disassembly of the trimer back into inert monomers and re‐establishes the repressive state, effectively shutting down the transcriptional response and ensuring that the HSR is active only as long as it is needed. Thus, the HSR is not merely a transient response to protein misfolding but a dynamic proteostasis network whose molecular chaperones can directly influence the stability, activity, and degradation of stress‐responsive transcription factors. This regulatory architecture provides an important mechanistic basis for its functional crosstalk with the NRF2 pathway.

## 4. Molecular Mechanisms of NRF2‐HSP Crosstalk

An expanding body of research reveals a deeply integrated network where the NRF2 and HSR pathways engage in extensive bidirectional crosstalk [17, 73, 74]. This interconnectivity is biologically logical, given that the instigating stressors are often intrinsically linked; for example, excessive ROS can lead to protein unfolding and damage, thereby inducing proteotoxic stress [75]. The communication between the NRF2 and HSR systems occurs at multiple levels, including the coregulation of gene transcription [74], the posttranslational modulation of pathway components [76], and the utilization of shared molecular hubs and protein degradation machinery [77].

### 4.1. Transcriptional Coregulation and the Nonuniversal Nature of the HSF1 Promoter Loop

One of the most direct forms of communication occurs at the genomic level through the reciprocal regulation of master transcription factors and their target genes. Compelling evidence has shown that NRF2 can directly potentiate the HSR by binding to a functional ARE located in the promoter region of the HSF1 gene itself [17]. This mechanism allows an oxidative or electrophilic challenge not only to trigger an antioxidant defense but also to proactively ’prime’ the cell for a more robust HSR, representing a sophisticated anticipatory stress response. The crosstalk also manifests as a convergence on specific stress gene promoters. A prime example is HMOX1, also known as HSP32, whose promoter contains both AREs and HSEs [78, 79]. This dual regulation makes HMOX1 a central effector gene that either NRF2 or HSF1 can robustly induce, allowing the cell to integrate signals from distinct stress types into a unified cytoprotective output [79]. However, critical evaluation reveals that NRF2‐driven activation of HSF1 is not a universal cellular phenomenon across all tissues but is highly context‐ and cell‐type‐dependent. In a seminal study, sodium arsenite‐induced oxidative stress stimulated an eight‐fold greater transcription of HSF1 in HT1080 fibrosarcoma cells compared to normal kidney epithelial (NKE) cells [17]. This tissue specificity is largely governed by the cell’s endogenous, basal ROS threshold and metabolic state. Tissues naturally exposed to high environmental stress (e.g., the gastrointestinal epithelium, lungs, and liver) maintain highly inducible and active NRF2‐ARE networks [17]. In contrast, normal lymphoid and hematopoietic cells display a highly restricted NRF2 response, with many canonical target genes remaining completely uninduced under an electrophilic challenge [3]. Thus, the coupling of NRF2 activation to HSF1 transcription is restricted to tissues with active redox‐sensing machinery and sufficient endogenous ROS to license the BRG1‐mediated chromatin remodeling required for HSF1 promoter activation [17].

### 4.2. Direct vs. Indirect Chaperone–Client Interactions

To understand the NRF2–HSP axis, it is important to distinguish direct physical interactions from indirect, cell context‐dependent regulatory pathways. Multiple experimental approaches, including yeast two‐hybrid screening, NMR spectroscopy, and mammalian coimmunoprecipitation, have provided evidence for direct interactions between NRF2, KEAP1, and components of the cytosolic HSP90 chaperone machinery [18, 80]. HSP90 has been reported to interact with NRF2, including through the Neh2 domain, potentially contributing to the maintenance of NRF2 protein stability and conformation [18, 20, 80, 81]. Similarly, direct interaction between NRF2 and the small HSP HSP27 has been reported in the nucleus. Type I phosphatidylinositol phosphate kinase (PIPKI) can transfer phosphatidylinositol 4,5‐bisphosphate (PtdIns(4,5)P_2_) to NRF2, promoting a conformational state that facilitates HSP27 binding to the Neh2 domain. This interaction may stabilize NRF2 and limit its degradation through a KEAP1‐independent mechanism [82, 83]. Collectively, these findings indicate that chaperone proteins can establish direct molecular links with NRF2 and thereby contribute to the regulation of its stability and transcriptional activity, although the functional consequences of these interactions may vary across cellular contexts.

In contrast, indirect and cell context‐dependent interactions are strongly influenced by the broader cellular signaling environment. A notable example involves the GSK‐3β/β‐TrCP degradation pathway. GSK‐3β has been reported to associate with HSP90‐containing chaperone complexes, and inhibition or depletion of HSP90 can destabilize GSK‐3β, potentially reducing its capacity to phosphorylate NRF2 at the Neh6 domain [31, 84, 85]. Because GSK‐3β‐mediated phosphorylation facilitates subsequent recognition of NRF2 by β‐TrCP and promotes KEAP1‐independent ubiquitination and degradation, HSP90 may indirectly influence NRF2 stability through the regulation of GSK‐3β [31, 84, 85]. Importantly, the functional relevance of this pathway is likely to depend on the cellular signaling context. For example, in cancer cells with constitutive activation of the PI3K/AKT pathway, AKT‐mediated phosphorylation can inhibit GSK‐3β activity, potentially attenuating the contribution of the HSP90/GSK‐3β axis to NRF2 regulation [18, 31, 84, 86]. Thus, although direct chaperone‐mediated interactions provide an important molecular interface between the HSP and NRF2 systems, downstream regulatory effects are shaped by the specific oncogenic, metabolic, and signaling characteristics of individual cell types.

### 4.3. Circumstances and Biochemical Stressors Driving Pathway Uncoupling

Although NRF2 and the HSR coordinate a joint cytoprotective front, they actively uncouple—displaying divergent or even opposing activities—under specific biochemical circumstances:

1. Opposing Roles in Autophagy and Cargo Degradation: While NRF2 acts as a transcriptional driver of selective autophagy by inducing the cargo receptor p62/SQSTM1 and LC3B, HSF1 exerts a direct, negative brake on autophagic flux [16]. Under bis(2‐hydroxybenzylidene) acetone (HBB2)‐induced stress, NRF2‐deficient cells exhibit a complete collapse of autophagic flux, whereas HSF1 can negatively regulate autophagic flux under specific stress conditions [16]. These findings provide evidence for functional uncoupling between NRF2‐ and HSF1‐dependent stress programs under this specific experimental condition.
2. Nutrient and Amino Acid Deprivation: During severe methionine deprivation, the cells encounter a proteostatic crisis that would conventionally require HSF1. However, HSF1 transcriptional activity is inactivated due to a lack of translational capacity and metabolic suppression [14, 17, 87]. Under these precise conditions, NRF2 actively uncouples from HSF1, directly binding to the HSP70 (HSPA1A) promoter to drive its transcription in an HSF1‐independent manner [17]. Thus, under amino acid starvation, NRF2 compensates for HSF1 to sustain chaperone defenses.
3. Differential Stress Thresholds and Cysteine Sensitivity: The KEAP1‐NRF2 pathway is chemically tuned to ultralow levels of electrophiles due to the exceptionally low pKa and high nucleophilicity of Cys151 on KEAP1, enabling rapid activation under mild, subtoxic oxidative stress [10, 88]. In contrast, HSF1 activation requires a significantly higher concentration threshold of electrophilic or thermal stress, which is necessary to cause the physical denaturation of chaperone complexes or induce the direct intermolecular disulfide bonding (Cys36–Cys103) required for HSF1 trimerization [57, 62]. Consequently, under low‐grade chronic physiological stress, the NRF2 and HSR pathways are temporally and concentrationally uncoupled, with only the NRF2 arm being active.

### 4.4. Shared Molecular Hubs: p62/SQSTM1 and the Proteasome

The NRF2 and HSR pathways physically and functionally converge on central cellular hubs that are critical for integrating stress responses [89]. The multifunctional adaptor protein p62/SQSTM1 stands out as the quintessential bridge linking autophagy, proteostasis, and the NRF2 antioxidant response [42]. p62 contains a KIR that directly competes with NRF2 for binding to the same pocket on Keap1, leading to robust, noncanonical NRF2 activation, especially when p62 levels accumulate due to impaired autophagy [90]. This interaction forms a powerful positive feedback loop, as the SQSTM1 gene is a direct transcriptional target of NRF2, thus allowing the cell to amplify both its protein clearance and antioxidant capabilities in a coordinated fashion [91]. Both pathways are also inextricably linked to the ubiquitin‐proteasome system (UPS) [92–94]. The HSR relies on the proteasome to degrade irreversibly damaged proteins that have been polyubiquitinated [95]. The crosstalk is further reinforced by the finding that NRF2 activation directly enhances the cell’s proteolytic capacity by transcriptionally upregulating the expression of key proteasome subunit genes, such as PSMB5 [84, 96]. This creates a synergistic loop where NRF2 not only manages oxidative stress but also boosts the machinery needed by the HSR to clear damaged proteins.

## 5. Functional Consequences of NRF2‐HSP Crosstalk in Cellular Physiology

The intricate molecular crosstalk between the NRF2 and HSR pathways translates into coordinated functional effects that enhance cellular resilience [74, 97]. Rather than functioning as isolated stress‐response systems, these pathways integrate antioxidant defense with proteostasis control, enabling cells to coordinate the detoxification of reactive species, maintenance of protein quality, and removal of damaged cellular components [84]. This functional integration is particularly important under conditions in which oxidative and proteotoxic stress occur simultaneously.

### 5.1. An Integrated and Fortified Stress Response

Most biologically relevant stressors, such as I/R or exposure to environmental toxins, are multifactorial, simultaneously inducing both oxidative and proteotoxic damage. The integrated NRF2‐HSR network is exceptionally well‐suited to manage such complex challenges. In cellular contexts in which NRF2‐dependent regulation of HSF1 is operative, NRF2‐mediated transcriptional upregulation of HSF1 may function as a proactive, anticipatory mechanism that primes the cell for a more effective HSR before proteotoxic damage becomes overwhelming [17]. This synergy is powerfully demonstrated in the realm of protein quality control [3]. In this coordinated system, HSP chaperones provide major components of the protein folding and triage machinery, facilitating refolding or directing irreversibly damaged proteins toward degradation. In parallel, NRF2 enhances cellular proteolytic capacity by supporting proteasome function and autophagy‐related pathways, thereby complementing chaperone‐mediated protein quality control [98]. This collaborative effort creates an exceptionally efficient pipeline for clearing toxic proteins and maintaining proteostasis.

### 5.2. Preservation of Mitochondrial Homeostasis

Mitochondria function as a critical hub for the NRF2‐HSR crosstalk, as these organelles are both a primary source of cellular ROS and are highly susceptible to proteotoxic stress [99]. The integrated network provides a multi‐pronged shield to maintain mitochondrial integrity and function. The HSR contributes to mitochondrial proteostasis through a dedicated network of mitochondrial chaperones, including mtHSP70, which participates in mitochondrial protein import and folding, and mtHSP60, which facilitates the folding and assembly of imported proteins within the mitochondrial matrix [100–102]. Concurrently, the NRF2 pathway provides a complementary defense by bolstering the mitochondrial antioxidant system through the upregulation of enzymes like manganese superoxide dismutase (SOD2) [103, 104]. Furthermore, NRF2 is a key regulator of mitochondrial quality control, promoting the selective removal of damaged mitochondria via mitophagy, thereby preventing the accumulation of dysfunctional, ROS‐generating organelles [99, 105]. This coordinated regulation is illustrated by studies showing that loss of either NRF2 or HSF1 results in altered mitochondrial membrane potential, compromised ATP production, and a significant increase in mitochondrial‐derived ROS, which further damages the cell if chaperone and antioxidant pathways are not coactivated [2, 12].

### 5.3. Potentiation of Anti‐Inflammatory and Antiapoptotic Programs

A crucial functional consequence of the NRF2‐HSR integration is the potentiation of pro‐survival cellular programs through the coordinated suppression of inflammation and apoptosis [2, 106]. Both pathways exert powerful anti‐inflammatory effects, in large part by converging on the negative regulation of the NF‐κB signaling pathway, a master regulator of inflammation [107]. NRF2 can transcriptionally repress pro‐inflammatory cytokine genes [108, 109], while HSP70 has been shown to interfere with key kinases in the NF‐κB activation cascade, providing a robust, dual brake on inflammatory signaling [110–112]. Similarly, the network creates a formidable antiapoptotic shield to protect cells from premature death. HSPs are well known for their ability to inhibit apoptosis at multiple checkpoints, for instance, by directly and indirectly interfering with caspase activation, preventing cytochrome c release from mitochondria, and binding to pro‐apoptotic factors such as Bax [113]. This is complemented by NRF2’s antioxidant program, which mitigates the upstream oxidative stress that often serves as a primary trigger for initiating the apoptotic cascade [114]. This combined action ensures that under transient or moderate stress, the default apoptotic pathway is actively held in check, giving the cell sufficient time to repair damaged proteins and DNA.

## 6. The Dual Role of NRF2‐HSP Crosstalk in Human Disease

Given its central role in orchestrating cellular resilience, the dysregulation of the NRF2‐HSP network is a key pathogenic factor in a wide spectrum of human diseases [52, 115, 116]. The NRF2 and HSP pathways individually operate as quintessential double‐edged swords [53, 117]. In many chronic conditions associated with aging, a progressive decline in the network’s capacity leads to increased cellular vulnerability and disease progression [1, 118, 119]. Conversely, in the context of malignancy, the very same cytoprotective pathways are frequently hijacked and constitutively hyperactivated by cancer cells to support their survival, proliferation, and resistance to therapy [3, 17, 74] (Table 2).

**Table 2:** Context‐dependent roles and therapeutic implications of NRF2–HSP crosstalk across major disease settings.

Disease context	NRF2 status	HSF1/HSP status	NRF2–HSP crosstalk mechanism	Major consequence	Therapeutic implication
Neurodegeneration	Reduced responsiveness/insufficient adaptive activation	Impaired stress responsiveness	Reduced antioxidant–proteostasis coordination	Proteostasis failure, oxidative injury	Restore NRF2/HSF1 activity
Cancer	Frequently constitutively activated	Frequently hyperactivated/overexpressed	Enhanced redox/proteostasis buffering	Survival, metabolic adaptation	Target NRF2/HSP network
I/R injury	Acute activation with ER‐stress‐associated constraint	Activated as part of the acute stress response	ER‐stress/XBP1–HRD1 signaling promotes KEAP1‐independent NRF2 degradation	ROS accumulation, tissue injury	Target XBP1–HRD1
Tumor immunity	Frequently hyperactivated	Context‐dependent	NRF2‐dependent immunometabolic regulation and HSP‐mediated chaperone signaling	TAM immunosuppression, altered T‐cell function, and immune evasion	Cell‐ and compartment‐specific targeting
Aging	Reduced transcriptional responsiveness	Reduced HSF1 activity and stress responsiveness	Age‐associated decline in NRF2 transcriptional responsiveness and HSF1 proteostasis capacity	Loss of cellular resilience	Restore stress adaptation

*Note*: The direction and magnitude of NRF2–HSP pathway activity are highly dependent on cell type, tissue, disease stage, and stress intensity. “Activation” refers to increased pathway activity or target‐gene expression, whereas “responsiveness” refers to the capacity of cells to mount an appropriate transcriptional response to stress. In ischemia–reperfusion injury, the XBP1–HRD1‐mediated constraint on NRF2 described here is supported particularly by evidence from renal I/R models and should not be generalized to all I/R settings. NRF2, nuclear factor erythroid 2–related factor 2; HSF1, heat shock factor 1; HSP, heat shock protein; ER, endoplasmic reticulum; I/R, ischemia–reperfusion; TAM, tumor‐associated macrophage; XBP1, X, box binding protein 1; HRD1, HMG‐CoA reductase degradation protein 1; KEAP1, Kelch‐like ECH, associated protein 1.

### 6.1. Neurodegenerative Diseases: Failure of Proteostasis and Redox Defenses

In neurodegenerative disorders, including Alzheimer’s, Parkinson’s, and Huntington’s diseases, progressive proteostasis failure and the accumulation of misfolded or aggregation‐prone proteins are major contributors to neuronal dysfunction and degeneration. In these conditions, enhancing NRF2–HSR crosstalk represents a critical therapeutic objective to promote aggregate clearance and mitigate oxidative damage [119–121]. Critical analysis of specific genetic and disease models highlights the molecular boundaries of this adaptive defense network. In Huntington’s disease, the expression of mutant Huntingtin (mHTT) containing expanded polyglutamine tracts induces profound oxidative stress, mitochondrial dysfunction, and proteostasis failure, leading to neuronal vulnerability and progressive degeneration [122, 123]. In this context, dysregulated protein aggregation and impaired clearance mechanisms can influence NRF2 signaling through noncanonical regulatory mechanisms associated with altered KEAP1 homeostasis and aggregate‐associated stress responses [124, 125]. Although sustained NRF2 activation may initially provide a compensatory cytoprotective response by enhancing antioxidant and detoxification programs, chronic activation can become insufficient to restore neuronal homeostasis because of progressive age‐dependent deterioration of cellular stress adaptation mechanisms [126].

Aging further restricts the efficiency of this protective network by impairing the transcriptional capacity of stress‐responsive pathways. In neural stem and progenitor cells (NSPCs), aging is associated with attenuation of NRF2‐dependent antioxidant gene induction despite preserved NRF2 nuclear localization, suggesting a decline in transcriptional responsiveness rather than a simple failure of NRF2 activation [92, 118, 127]. This limitation is further compounded by the age‐related disruption of HSF1 regulation. The NAD^+^‐dependent deacetylase SIRT1 maintains HSF1 in a transcriptionally competent state by preventing excessive acetylation. Conversely, reduced SIRT1 activity promotes p300/CBP‐mediated HSF1 acetylation, impairing HSF1 binding to HSEs, accelerating HSF1 attenuation, and reducing chaperone gene expression [14, 128]. Therefore, in neurodegenerative conditions, the therapeutic window for enhancing cellular stress resistance is constrained by the simultaneous decline of both NRF2‐mediated antioxidant defense and HSF1‐driven proteostasis programs.

### 6.2. Cancer: The Dark Side of Cytoprotection, Metabolic Reprograming, and Therapeutic Vulnerabilities

In stark contrast to neurodegeneration, the pathology of cancer frequently involves the hijacking and constitutive activation of the NRF2–HSR/HSP axis [17]. Cancer cells endure persistent intrinsic stress due to elevated metabolic flux, oncogene‐driven ROS production, and proteome instability, rendering them “addicted” to cytoprotective networks for survival. Indeed, NRF2 is frequently constitutively activated in a broad spectrum of solid tumors, often via somatic alterations in NFE2L2 or its primary repressor KEAP1, disrupting the normal regulation of proteotoxic and oxidative stress responses [129, 130]. Concurrently, the HSR is frequently upregulated in malignancies: molecular chaperones such as HSP90 and HSP70 are overexpressed and play critical roles in stabilizing mutated or overexpressed oncoproteins, buffering folding stress, and supporting tumor growth [131, 132]. Somatic loss‐of‐function mutations in KEAP1 or activating mutations in NRF2 that disrupt KEAP1 recognition, including alterations affecting the DLG and ETGE motifs, are highly enriched in non‐small‐cell lung cancer (NSCLC), esophageal squamous cell carcinoma, and head and neck cancers [88, 133]. This constitutive activation drives extensive metabolic reprograming, re‐directing glucose and glutamine flux toward anabolic pathways. NRF2 directly upregulates G6PD, malic enzyme 1 (ME1), and transketolase (TKT) [134], driving the PPP to produce the NADPH required for lipid synthesis, nucleotide production, and GSH recycling [88]. Furthermore, NRF2 upregulates UDP‐glucose dehydrogenase (UGDH), channeling UDP‐glucose into UDP‐glucuronic acid (UDP‐GlcA) [88, 135]. This metabolic detour creates a synthetic‐lethal vulnerability in KEAP1‐mutant tumors: loss of UDP‐xylose synthase 1 (UXS1) leads to excessive accumulation of UDP‐GlcA, which can disrupt Golgi homeostasis and receptor trafficking and, in KEAP1‐mutant cells with high NRF2‐driven UGDH expression, deplete pyrimidine nucleotide pools and induce replication stress [88, 135]. This demonstrates that while the NRF2‐HSP axis provides a formidable cytoprotective shield, it simultaneously introduces unique metabolic vulnerabilities that can be exploited therapeutically.

### 6.3. I/R Injury: Cytoprotection and Stress‐Induced Constraints

Although chronic activation of the NRF2 pathway is widely implicated in tumorigenesis, the acute and robust induction of the NRF2–HSR/HSF1 axis is profoundly cytoprotective in settings of I/R injury [14, 136, 137]. In models of myocardial, cerebral, and renal I/R injury, coordinated activation of NRF2 and HSRs attenuates reperfusion‐associated oxidative and proteotoxic stress, limits tissue injury, and improves functional recovery [137–139]. On a broader scale, a progressive decline in the functional capacity of both NRF2‐mediated antioxidant defenses and HSF1‐dependent proteostasis is increasingly recognized as an important contributor to biological aging [118, 120, 121, 140]. However, critical biochemical evaluation reveals a major bottleneck in this response under severe ischemic conditions. I/R is characterized by intense endoplasmic reticulum (ER) stress (ERS). In renal I/R injury specifically, however, ER‐stress‐induced XBP1–HRD1 signaling has been shown to impose an additional constraint on NRF2 activity. Under ERS, the E3 ubiquitin ligase HRD1, which is localized in the ER membrane, is transcriptionally upregulated via the XBP1 pathway [141]. HRD1 directly binds to the Neh4 and Neh5 transactivation domains of NRF2, targeting it for ubiquitin‐proteasome‐mediated degradation independently of KEAP1 [141]. This HRD1‐mediated NRF2 degradation acts as a negative regulatory checkpoint that depletes the cell’s cytoprotective antioxidant pool during the critical transition from I/R, precipitating a burst of mitochondrial ROS and subsequent cell death [98]. Consequently, therapeutic strategies to inhibit the XBP1–HRD1 axis or prevent HRD1‐mediated NRF2 degradation represent highly targeted approaches to restore redox‐proteostasis cooperation during myocardial or renal I/R injury [45, 142].

### 6.4. NRF2‐HSP Crosstalk in Tumor Immunity and Immunometabolism

The KEAP1–NRF2–HSP axis acts as a critical regulator of immune cell immunometabolism, macrophage plasticity, and adaptive T‐cell responses within the hostile microenvironment of solid tumors. Recent high‐definition spatial transcriptomics, single‐cell RNA sequencing, and genetic models have demonstrated that NRF2 activation has emerged as an important regulator of immunosuppressive phenotypes in the tumor stroma [143, 144].

#### 6.4.1. Regulation of Immune‐Cell Metabolism

Within the tumor microenvironment, the metabolic programing of infiltrating immune cells is tightly governed by cellular stress pathways [143]. During activation, effector T cells undergo metabolic reprograming to meet the bioenergetic demands of clonal expansion, generating elevated ROS as a byproduct [145]. In regulatory T cells (Tregs), NRF2 activation significantly enhances glucose uptake and upregulates mammalian target of rapamycin (mTOR) activity, thereby modifying cellular survival and proliferative capacity under metabolic stress [145]. In invariant natural killer T (iNKT) cells, NRF2 activation promotes mitochondrial biogenesis and glucose transport via GLUT1 upregulation [145]. This stress‐responsive metabolic control is intrinsically coupled with the HSR. The molecular chaperone HSP90 directly interacts with and stabilizes hypoxia‐inducible factor 1‐alpha (HIF‐1α) and AKT, protecting them from proteasomal degradation [146]. This stabilization preserves the activity of the PI3K/AKT signaling pathway, which is indispensable for the glycolysis‐dependent activation and survival of effector T cells [146]. Furthermore, HSP90 interacts with pyruvate kinase M2 (PKM2), facilitating its translocation into the nucleus to coactivate HIF‐1α and STAT3, which drives the glycolytic switch in inflammatory macrophages [146]. Additionally, the metabolic‐inflammatory feedback loop is regulated by the mitochondrial‐derived immunometabolite itaconate, which alkylates specific KEAP1 cysteine sensors (Cys151, Cys273, and Cys288) to activate NRF2 [145, 147]. This endogenous metabolic “off‐switch” suppresses the transcription of pro‐inflammatory cytokines such as IL‐6 and IL‐1β in macrophages, demonstrating how the NRF2‐HSP axis integrates Krebs cycle intermediates with cellular proteostasis and redox signaling to control immune cell fate [108, 145, 147, 148]. Thus, NRF2‐mediated redox control and HSP90‐dependent stabilization of metabolic signaling proteins converge to sustain the metabolic fitness of immune cells exposed to nutrient limitation, hypoxia, and oxidative stress.

#### 6.4.2. Macrophage Polarization and Tumor‐Associated Macrophages (TAMs)

TAMs exhibit high plasticity, existing along a functional spectrum between pro‐inflammatory (immunogenic) and anti‐inflammatory (suppressive) states. Recent spatial transcriptomic and single‐cell transcriptomic studies have revealed that immunotherapy‐induced or radiotherapy‐induced necrosis drives a highly structured spatial zonation of TAMs [143]. While pro‐inflammatory Cxcl9+ macrophages accumulate at the tumor margins to recruit effector T cells, anti‐inflammatory, immunosuppressive Spp1+/Arg1+ macrophages heavily cluster in perinecrotic regions characterized by severe hypoxia and cellular stress [143]. This spatial‐functional dichotomy is directly driven by a gradient of NRF2 activation [143]. Under necrotic stress, ischemia, or exposure to heme, NRF2 is stabilized and translocates to the nucleus of TAMs [143]. Although this NRF2 activation is cytoprotective, it concurrently acts as a molecular brake on macrophage plasticity, locking them into an anti‐inflammatory, NRF2‐imprinted stress‐TAM state [143]. These NRF2‐imprinted TAMs silence critical IFN‐STAT1 signaling pathways, downregulate MHC‐II, and suppress the expression of T‐cell‐recruiting chemokines such as CXCL9 and CXCL10 [143]. Consequently, they fail to prime or expand CD8+ T cells, thereby driving tumor cell invasion, epithelial–mesenchymal transition, and metastasis [143]. Similarly, epithelial NRF2 deficiency in esophageal squamous cell carcinoma remodeled the myeloid compartment, promoting a selective phenotypic shift from Folr2+ tissue‐resident macrophages to Mrc1+ macrophages, which reorganizes the myeloid landscape and reduces CXCL1/2/3‐enriched myeloid‐driven immunosuppressive recruitment signals [144]. Furthermore, target‐specific interventions like inhibiting GPX4 or xCT in TAMs can promote lipid peroxidation and trigger metabolic reprograming from oxidative phosphorylation to glycolysis, successfully reversing the pro‐tumoral M2 phenotype to an active, tumoricidal M1 state [147].

#### 6.4.3. T‐Cell Function and Adaptive Immunity

The maintenance of adaptive antitumor immunity is highly dependent on redox‐ and chaperone‐mediated signaling within T lymphocytes. NRF2 restricts CD8+ T‐cell function through the regulation of GSH and cysteine availability [149]. Because early‐phase activated T cells are incapable of synthesizing sufficient GSH, they rely on antigen‐presenting cells such as macrophages to supply these precursors; macrophages lacking NRF2 exhibit decreased expression of the cystine/glutamate transporter xCT and GCLM, thereby failing to support T‐cell activation and priming [150]. Conversely, persistent NRF2 hyperactivation can suppress effector T‐cell differentiation. Pharmacological NRF2 activation by tert‐butyl hydroquinone (tBHQ) has been shown to repress the production of interferon‐gamma (IFN‐γ) while promoting a Th2‐skewed cytokine profile in CD4+ T cells [145, 151]. Furthermore, NRF2 activation in Tregs suppresses the differentiation of pro‐inflammatory Th17 cells and alters peripheral tolerance [145, 151]. Importantly, the effects of NRF2 on lymphocyte biology are highly cell‐state‐dependent; NRF2 may support metabolic fitness and survival in Tregs, while excessive or persistent NRF2 activity can constrain effector T‐cell differentiation and function. In parallel, molecular chaperones and HSF1 exert profound context‐dependent control over T‐cell function. Extracellular HSPs, such as HSP70, act as powerful immunogens and “chaperokines” [113, 152]. Extracellular HSP70 binds to scavenger receptors (e.g., LOX‐1 and CD91) on APCs, enhancing the cross‐presentation of tumor‐associated antigenic peptides to MHC class I and class II molecules, which robustly stimulates CD8+ cytotoxic and CD4+ helper T lymphocyte responses [113, 146, 153]. Although extracellular HSPs represent a distinct immunological arm of HSP biology, their immunomodulatory effects should be considered separately from the intracellular chaperone functions that directly intersect with NRF2 signaling. However, within the immunosuppressive tumor microenvironment, HSF1 can directly undermine T‐cell surveillance. Activated HSF1 in NK cells downregulates the expression of cytotoxic and effector‐associated markers, including NK1.1 and IFN‐γ [59, 154]. Moreover, tumor‐intrinsic HSF1 actively represses the secretion of CCL5 in breast cancer cells, thereby blocking CD8+ T‐cell recruitment and creating an immunologically cold niche [59].

#### 6.4.4. Tumor Immune Evasion and the Tumor Microenvironment

Constitutive hyperactivation of the NRF2‐HSP axis represents a major pathway of tumor immune exclusion and immunotherapy resistance. Genomic characterization of solid tumors shows that KEAP1/NRF2‐mutant malignancies are strongly enriched in the “immune desert” phenotype, characterized by a low number of tumor‐infiltrating lymphocytes (TILs) and poor T‐cell invasion [85, 155]. Mechanistically, NRF2 hyperactivation directly suppresses the type‐I interferon response. Mechanistically, NRF2 activation suppresses the type I interferon response by reducing the stability of stimulator of interferon genes (STING; TMEM173) mRNA, thereby attenuating downstream type I interferon production and potentially contributing to tumor immune evasion [85, 147, 148]. Electrophilic metabolites such as itaconate derivatives can further promote this immunosuppressive axis through NRF2 activation. Furthermore, NRF2 and its downstream targets directly regulate the expression of immune checkpoints and immunosuppressive enzymes. Ultraviolet radiation and oxidative stress upregulate the expression of programed death ligand 1 (PD‐L1) in an NRF2‐dependent manner, mediating T‐cell exhaustion and immune escape [85, 147]. Additionally, the coordinated activity of NRF2 and ATF4 under ER stress transcriptionally induces cyclooxygenase 2 (COX2), encoded by the PTGS2 gene [147]. The resulting overproduction of prostaglandin E2 (PGE2) attenuates T‐cell receptor (TCR) signaling and blocks the infiltration of type‐I dendritic cells (DCs) into the tumor parenchyma, establishing profound immune tolerance [147]. While extracellular HSPs can trigger immune surveillance, intracellular HSP70 and HSP90 function as cytoprotective shields that buffer the proteotoxic and genotoxic stress of malignant cells, enabling them to survive granzyme‐mediated apoptosis and escape immune clearance [119, 156, 157]. Collectively, these disease contexts illustrate the context‐dependent nature of NRF2–HSP crosstalk, in which insufficient stress response capacity promotes cellular vulnerability, whereas persistent or dysregulated activation can support tumor survival, metabolic adaptation, and immune evasion.

## 7. Therapeutic Targeting of the NRF2‐HSP Axis

The dual and context‐dependent role of the NRF2–HSF1–HSP network in controlling cellular fate renders it a highly tractable therapeutic axis [10, 85, 158]. Therapeutic strategies targeting this axis are fundamentally context‐dependent: in degenerative, inflammatory, and acute stress‐related diseases, the therapeutic objective is generally to restore NRF2‐mediated cytoprotection or enhance proteostasis, whereas in cancer, strategies are increasingly focused on selectively disrupting NRF2 signaling or HSR components, such as HSF1 and HSP90, in tumor cells that are dependent on these stress‐adaptive networks [159–162].

### 7.1. Synergistic Activation for Cytoprotective Therapies

In chronic or degenerative diseases where the failure of the NRF2–HSF1–HSP axis underlies pathology, the therapeutic objective is to restore or amplify its cytoprotective capacity [160, 163]. This therapeutic principle has clinical precedent: dimethyl fumarate (DMF), a well‐characterized electrophilic NRF2 activator, is approved for multiple sclerosis, where its efficacy is attributed to potent antioxidant and anti‐inflammatory mechanisms in the central nervous system [160, 164, 165]. DMF covalently modifies KEAP1 Cys151, leading to NRF2 accumulation and transcriptional induction of NQO1 and HMOX1 (HO‐1) [4, 164]. Bardoxolone methyl (CDDO‐Me) has demonstrated NRF2‐dependent effects on renal function in clinical studies of Alport syndrome and diabetic kidney disease, although its development has been constrained by important safety concerns, particularly fluid retention and cardiovascular adverse events [166, 167]. On the proteostasis front, coinducers of the HSR such as arimoclomol, which enhance HSF1 transcriptional activity specifically under proteotoxic stress to upregulate HSP70 and HSP27 expression, have reached clinical evaluation (e.g., in amyotrophic lateral sclerosis and Niemann‐Pick disease type C), showcasing the therapeutic feasibility of target activation [168, 169].

### 7.2. Coinhibition Strategies for Cancer Therapy

In oncology, a promising therapeutic strategy is to disrupt the cytoprotective network formed by the NRF2–HSF1–HSP90 axis, which malignant cells exploit to survive therapeutic stress [170, 171]. In particular, HSP90 inhibitors, which promote destabilization and proteasomal degradation of numerous oncogenic client proteins, represent one of the most extensively investigated approaches for disrupting malignant proteostasis [156, 158, 172]. Concurrently, direct pharmacological inhibition of NRF2 or disruption of the KEAP1–NRF2 axis remains predominantly at the preclinical stage, particularly for tumors harboring KEAP1 or NFE2L2 alterations that drive constitutive NRF2 activation and therapy resistance [85, 155, 173]. Emerging preclinical evidence suggests that cotargeting both NRF2 signaling and components of the HSR (HSP90 or HSF1) may create a synthetic lethal vulnerability in NRF2‐addicted cancer cells, thereby enhancing tumor sensitivity to standard therapies [171, 174, 175]. While this combination strategy offers a compelling rationale, its clinical translation will require careful optimization of dosing, scheduling, toxicity management, and predictive biomarker‐based patient selection.

### 7.3. Targeting the NRF2‐HSP Axis in the Tumor Immune Microenvironment

Given the bidirectional feedback loop between tumor cells and the immune stroma, therapeutic strategies targeting the NRF2‐HSP axis must be carefully calibrated to avoid detrimental systemic consequences. Preclinical studies indicate that systemic NRF2 inhibition can be a double‐edged sword; while NRF2 blockade sensitizes cancer cells to therapy, global NRF2 deletion in host myeloid cells exacerbates lung metastasis and accelerates tumor growth by increasing ROS accumulation in myeloid‐derived suppressor cells (MDSCs), which paralyzes CD8+ T‐cell immunity [143]. Therefore, therapeutic strategies should prioritize tumor‐ or myeloid‐compartment‐selective targeting rather than indiscriminate systemic NRF2 inhibition. Reversing NRF2 activity selectively in TAMs (such as via myeloid‐targeted nanoparticles or macrophage‐specific NRF2 ablation) preserves macrophage‐T‐cell cross‐talk and restores the CD8+ T‐cell‐mediated antitumor response, dramatically enhancing the efficacy of agonistic anti‐CD40 or anti‐PD‐1/PD‐L1 immunotherapies [143, 150].

Another highly translational strategy leverages the NRF2‐dependent transcriptome for tumor‐selective synthetic lethality. Since NRF2‐addicted cancer cells express high baseline levels of NQO1 and thioredoxin reductase 1 (TXNRD1), these enzymes can be exploited to bioactivate prodrugs specifically within malignant cells [155, 174]. Several geldanamycin‐derived HSP90 inhibitors, including tanespimycin (17‐AAG), alvespimycin (17‐DMAG), and retaspimycin (IPI‐504), have been investigated as NQO1‐bioactivated prodrugs, exploiting the elevated reductive capacity of NQO1‐high tumor cells [155, 174].

Furthermore, disrupting the tumor‐intrinsic NRF2‐GSH peroxidase 2 (GPX2) axis has emerged as a potent method to sensitize tumors to radiotherapy‐induced immunogenic cell death (ICD) [144]. Deletion or silencing of epithelial NRF2 suppresses GPX2, leading to a collapse of redox homeostasis and sensitizing cancer cells to ionizing radiation [144]. Upon radiotherapy, these NRF2‐deficient cells undergo immunogenic demise, characterized by the robust release of damage‐associated molecular patterns (DAMPs) such as HMGB1, extracellular ATP, and cell‐surface exposure of calreticulin (CRT) [144]. These DAMPs trigger DC maturation, upregulation of costimulatory molecules (CD80/CD83), and the secretion of CXCL10, which subsequently recruits and primes cytotoxic CD8+ T cells [144]. Collectively, these findings provide a rationale for investigating combinations of selective NRF2/HSP‐axis inhibitors with immunotherapy or radiotherapy as a strategy to convert immunologically “cold” tumors into more inflamed and therapy‐responsive microenvironments [144].

### 7.4. Translational Challenges, Pharmacodynamic (PD) Biomarkers, and Clinical Duality

The clinical translation of NRF2‐ and HSP‐targeted modulators faces substantial hurdles, chiefly a narrow therapeutic window and off‐target toxicities. Systemic pan‐inhibition of HSP90 or NRF2 disrupts fundamental homeostatic defenses in normal tissues, causing dose‐limiting toxicities [158, 172]. For instance, first‐generation geldanamycin‐derived HSP90 inhibitors, exemplified by 17‐AAG (tanespimycin), showed limited clinical development because of hepatotoxicity, poor solubility, and pharmacokinetic limitations, together with the induction of compensatory HSF1‐mediated HSRs [172, 176, 177]. Inhibiting HSP90 triggers the rapid release and trimerization of HSF1, inducing the compensatory upregulation of cytoprotective chaperones (HSP70 and HSP27), which actively buffers the tumor cell against apoptotic signals [172, 174, 178]. Similarly, the second‐generation nongeldanamycin HSP90 inhibitor Ganetespib (STA‐9090) failed in the Phase III GALAXY‐2 lung adenocarcinoma trial, failing to improve overall survival as a combination therapy [158, 179, 180]. These second‐generation pan‐inhibitors also introduced distinct ocular toxicities, including photopsia and blurred vision, due to retinal target engagement [180, 181]. On the NRF2 activation front, CDDO‐Me (Bardoxolone Methyl) completed Phase III but was terminated in Stage 4 CKD patients due to severe fluid retention and heart failure risks, highlighting the systemic dangers of uncalibrated NRF2 induction [182].

To overcome these barriers, modern clinical oncology focuses on three pillars of precision medicine: (1) Isoform‐Selective Inhibitors: The development of Pimitespib (TAS‐116/Jeselhy), approved in Japan in 2022 for refractory GIST, selectively targets cytosolic HSP90α and HSP90β while completely sparing mitochondrial TRAP1 and ER GRP94, thereby minimizing systemic and retinal toxicity [183, 184]. (2) Direct HSF1 transcription Inhibitors: Novel compounds like NXP800 (CCT361814) directly inhibit HSF1 Ser326 phosphorylation and nuclear translocation without triggering compensatory chaperone expression, currently progressing in Phase I/II trials for advanced solid tumors [58, 59, 185]. (3) Candidate and Emerging PD Biomarkers: Robust monitoring of target engagement is essential. Validated PD biomarkers include the measurement of NQO1 and AKR1C1 mRNA/protein levels in peripheral blood mononuclear cells (PBMCs) to confirm NRF2 activation [10, 171], the quantification of HSF1 Ser326 phosphorylation in biopsies to assess HSF1 inhibition [58, 185], and the surrogate monitoring of serum HSP70/HSP72 to confirm systemic chaperone modulation [178, 180]. Applying these molecular tools enables patient selection based on oncogenic dependencies (e.g., KEAP1 or NFE2L2 mutational status), turning a once‐unpredictable therapeutic approach into a precise, targeted intervention. A summary of representative pharmacological and experimental strategies targeting the NRF2–HSP axis, together with their mechanisms, clinical evidence, pharmacodynamic biomarkers, limitations, and potential patient‐selection approaches, is provided in Table 3.

**Table 3:** Therapeutic targeting of the NRF2–HSP axis: mechanisms, clinical evidence, biomarkers, and translational considerations.

Agent/strategy	Primary target/pathway	Mechanism of action	Disease/cancer context	Evidence and clinical status	Candidate pharmacodynamic/predictive biomarkers	Major limitations/toxicity	Potential combination/patient‐selection strategy	Ref.
Dimethyl fumarate (DMF)	KEAP1–NRF2	Electrophilic modification of KEAP1 → NRF2 stabilization and cytoprotective gene induction	Relapsing‐remitting multiple sclerosis (RRMS)	FDA‐approved for relapsing forms of multiple sclerosis; phase III evidence	NQO1, HO‐1; NRF2 transcriptional signature	Flushing, GI effects, lymphopenia; rare PML	Monitor lymphocyte counts; relevant where NRF2 activation is therapeutically beneficial	[186–188]
Sulforaphane (SFN)	KEAP1–NRF2	Electrophilic activation of NRF2 → induction of antioxidant/cytoprotective genes	Cancer prevention; COPD; metabolic and neurodegenerative disorders	Clinical investigation; not approved as an NRF2‐directed therapy; variable human responses	NQO1, HO‐1, AKR1C1, AKR1C3	Variable bioavailability; inconsistent clinical target engagement	Potential chemopreventive/cytoprotective adjunct; NRF2 responsiveness requires validation	[10, 189]
Bardoxolone methyl (CDDO‐Me)	KEAP1–NRF2	Potent electrophilic NRF2 activation with modulation of inflammatory signaling	CKD; diabetic kidney disease; Alport syndrome	Advanced clinical development; BEACON terminated for cardiovascular safety	eGFR; oxidative/inflammatory biomarkers	Fluid retention, hypertension and cardiovascular risk	Requires careful cardiovascular risk stratification	[10, 182]
Arimoclomol	HSF1/heat‐shock response	Amplifies stress‐induced HSF1 activity and chaperone expression	Niemann–Pick disease type C (NPC); previously investigated in ALS	FDA‐approved (2024) with miglustat for neurological manifestations of NPC	NPCCSS; proteostasis‐related biomarkers	Generally acceptable tolerability; adverse events require monitoring	Genetically confirmed NPC; used with miglustat	[168, 190]
Tanespimycin (17‐AAG)	HSP90	ATP‐competitive HSP90 inhibition → degradation of client oncoproteins	Solid tumors; hematological malignancies	Clinical development; not approved; limited antitumor activity	HSP70 induction; HSP90‐client depletion	Hepatotoxicity, thrombocytopenia, GI effects; formulation limitations	Potentially relevant to HSP90‐client‐dependent tumors; combinations investigated	[176, 177]
Ganetespib	HSP90	HSP90 inhibition → destabilization of multiple oncogenic client proteins	Advanced NSCLC and other malignancies	Phase III failure; GALAXY‐2 stopped for futility	HSP70 induction; client‐protein degradation	Limited efficacy; treatment‐related toxicity; lack of validated predictive biomarkers	Molecular selection and validated PD biomarkers remain unresolved	[179]
Pimitespib (TAS‐116)	Cytosolic HSP90α/β	Selective inhibition of cytosolic HSP90 → disruption of oncogenic client‐protein stability	Advanced GIST after standard TKI failure	Approved in Japan; phase III demonstrated improved PFS	Clinical response/PFS; HSP90‐client modulation	Diarrhea, decreased appetite; generally limited ocular toxicity	Selected for GIST refractory to imatinib, sunitinib and regorafenib	[183, 184, 191]
HSP70 inhibitors/modulators (VER‐155008, YK‐5)	HSP70 family	Inhibition of HSP70 chaperone activity and stress adaptation	Proteotoxic‐stress‐dependent cancers	Preclinical/early translational	HSP70 activity; proteotoxic stress; apoptosis	Narrow therapeutic window; potential normal‐tissue toxicity	Combination with HSF1 inhibition may suppress compensatory HSP induction	[192]
HSF1 inhibitors (e.g., KRIBB11, DTHIB)	HSF1/HSR	Suppression of HSF1 transcriptional activity or promotion of active HSF1 degradation	Cancer, including prostate and colorectal cancer	Preclinical; no established clinical HSF1 inhibitor	HSF1 phosphorylation/nuclear abundance; HSP70/HSP90; HSF1 signature	Lack of clinically validated selective inhibitors; potential proteostasis toxicity	Potentially useful in HSF1‐high/HSP‐dependent tumors; combination strategies remain experimental	[193–195]
NRF2 inhibition/synthetic‐lethal targeting	NRF2‐dependent metabolic/proteostatic programs	Targets metabolic dependencies created by constitutive NRF2 activation	KEAP1‐mutant/NFE2L2‐altered cancers	Preclinical/emerging translational strategy	NRF2 signature; NQO1/TXNRD1; KEAP1/NFE2L2 genotype	Limited direct druggability; risk of disrupting normal stress adaptation	Select tumors with KEAP1 loss/NFE2L2 activation and high NRF2 activity	[88, 196, 197]
NRF2–HSR cotargeting	NRF2 + HSF1/HSP network	Simultaneous disruption of redox and proteostasis adaptation	NRF2‐ and/or HSP‐dependent cancers	Experimental concept; clinical efficacy unproven	NRF2 + HSF1/HSP signatures; ROS/proteotoxic stress	Potential systemic toxicity; no validated clinical biomarkers	Candidate tumors with constitutive NRF2 activation and high HSP dependence	[64, 180, 196]
HSP90 inhibition + proteasome inhibition	HSP90 + ubiquitin–proteasome system	Concurrent impairment of protein stabilization and degradation → severe proteotoxic stress	Multiple myeloma; proteostasis‐dependent cancers	Preclinical and clinical investigation	HSP70; ubiquitinated proteins; proteasome activity; apoptosis	Overlapping toxicity; compensatory HSR	Potentially relevant to tumors with high proteotoxic burden	[178, 180]

Abbreviations: CKD, chronic kidney disease; DMF, dimethyl fumarate; GIST, gastrointestinal stromal tumor; HO‐1, heme oxygenase‐1; HSF1, heat shock factor 1; HSP, heat shock protein; KEAP1, Kelch‐like ECH‐, associated protein 1; NFE2L2, nuclear factor erythroid 2‐like 2; NPC, Niemann–Pick disease type C; NQO1, NAD(P)H quinone oxidoreductase 1; NRF2, nuclear factor erythroid 2‐related factor 2; OS, overall survival; PFS, progression‐free survival; RRMS, relapsing‐remitting multiple sclerosis.

## 8. Conclusion

The NRF2 signaling pathway and the HSR constitute an interconnected stress‐adaptation network that coordinates redox homeostasis and proteostasis. Their crosstalk operates through multiple regulatory layers, including transcriptional coregulation, protein–protein interactions, and shared molecular hubs such as p62/SQSTM1 and the ubiquitin–proteasome system. This integrated network enables cells to simultaneously counteract oxidative and proteotoxic stress, preserve mitochondrial and cellular homeostasis, and promote survival under adverse conditions.

Importantly, the functional consequences of NRF2–HSP crosstalk are highly context‐ and stress‐dependent. Transient activation generally supports cytoprotection, tissue repair, and recovery from acute insults such as I/R injury, whereas impaired stress adaptation contributes to neurodegenerative and age‐related disorders. Conversely, persistent or constitutive activation can be exploited by malignant cells to sustain metabolic reprograming, proteostasis, immune evasion, and therapeutic resistance. The emerging evidence also highlights the complexity of this network within the tumor microenvironment, where NRF2 and HSP pathways can exert distinct and sometimes opposing effects on macrophage and T‐cell function.

These context‐dependent effects underscore the limitations of indiscriminate pharmacological modulation of the NRF2–HSP axis. Therapeutic activation may be beneficial in degenerative and inflammatory diseases, whereas selective inhibition of NRF2, HSF1, or HSPs may provide opportunities for cancer treatment. However, systemic modulation carries substantial risks, emphasizing the need for tissue‐, cell‐, and disease‐specific strategies.

Future clinical translation will therefore require molecular stratification, reliable pharmacodynamic biomarkers, advanced disease models, and rational combination therapies. Integrating these approaches with precision medicine may enable selective exploitation of NRF2–HSP crosstalk to enhance cytoprotection where beneficial while overcoming pathological stress adaptation in cancer and other diseases.

## Funding

No funding was received for this study.

## Conflicts of Interest

The authors declare no conflicts of interest.

## Data Availability

The data that support the findings of this study are available upon request from the corresponding author. The data are not publicly available due to privacy or ethical restrictions.
